# Systemic Therapy for Operable NSCLC: A Review of the Literature and Discussion of Future Directions

**DOI:** 10.3390/jcm14124127

**Published:** 2025-06-11

**Authors:** Matthew M. Mirsky, Katherine E. Myers, Sami O. Abul-Khoudoud, Joan Y. Lee, Debora S. Bruno

**Affiliations:** 1 University Hospitals Seidman Cancer Center, Cleveland, OH 44106, USA; katherine.myers@uhhospitals.org (K.E.M.); joan.lee2@uhhospitals.org (J.Y.L.); 2 Department of Solid Tumor Oncology, Thoracic Division, Case Western Reserve University, Cleveland, OH 44106, USA; 3 Department of Internal Medicine, Case Western Reserve University, Cleveland, OH 44106, USA; 4 Department of General Surgery, Case Western Reserve University, Cleveland, OH 44106, USA; sami.abul-khoudoud@uhhospitals.org; 5 Department of Radiation Oncology, Case Western Reserve University, Cleveland, OH 44106, USA; 6 Department of Thoracic Oncology, City of Hope Cancer Center, Atlanta, GA 30265, USA

**Keywords:** perioperative non-small cell lung cancer, targeted therapy, immune checkpoint inhibitor

## Abstract

Operable non-small cell lung cancer (NSCLC) has been traditionally managed with surgical resection, often followed by adjuvant chemotherapy with or without radiotherapy. However, disease recurrence still occurs approximately 50% of the time. Most recently, (neo) adjuvant/perioperative systemic therapy has evolved to include checkpoint inhibitor therapy and targeted therapies that have proved successful in advanced disease settings. We provide a comprehensive review of the trials investigating neoadjuvant, adjuvant, and perioperative systemic therapies in resectable lung cancer, including a discussion on surrogate survival endpoints. We review the management of N2 disease, the utility of circulating tumor DNA (ctDNA) in determining the risk and benefit from systemic therapy in operable NSCLC, as well as future directions of investigation.

## 1. Introduction

Non-small cell lung cancer (NSCLC) represents the second most common cause of cancer in the United States (U.S.), and the leading cause of cancer-related deaths in the U.S. and worldwide [1]. Historically, the management of early-stage NSCLC has traditionally revolved around surgical resection with curative intent. However, the 5-year disease-free survival (DFS) rate for stage II and III NSCLC is approximately 40% [2]. In patients who underwent surgical resection with curative intent, up to 30–70% will experience disease recurrence [3]. After NSCLC recurrence, 5-year overall survival (OS) ranges between 2 and 13% [4].

The high rate of recurrence and disappointing 5-year OS observed in patients who undergo surgical resection in early-stage NSCLC is hypothesized to be the result of baseline micro-metastasis, which are not captured by currently available staging techniques. One meta-analysis published in 2022 demonstrated that patients with stage I–IIIA NSCLC who had lymph node micro-metastases at the time of resection (identified by multiple sectioning, IHC, or RT-PCR) had significantly poorer OS and DFS compared to those without micro-metastatic disease [5].

Given the high rate of disease recurrence despite surgical resection, the role of systemic therapy is of particular interest in disease recurrence and survival. Mechanistically, systemic therapy is likely to improve survival outcomes by reducing and potentially eradicating the burden of micro-metastasis in the perioperative setting, as well as improving the success of surgical resection by reducing the overall tumor size, and a possible inhibition of growth factor stimulation to residual cancer cells induced by surgery and subsequent wound healing when delivered in neoadjuvant fashion [6]. Initial studies examining the role of chemotherapy in resectable NSCLC began to surface in the mid-1990s, with mixed OS results. Of note, two small, randomized studies demonstrated strongly positive results in favor of neoadjuvant therapy in addition to surgical resection in patients with stage IIIA disease [7,8]. However, both studies were stopped early, and the results were felt to be unreliable, as patients assigned to the control arm (resection only) fared worse than expected. The *MRC LU22/NVALT 2/EORTC 08012* study built upon these early studies using a randomized, multi-center design. Conducted across Europe between 1995 and 2007, the study ultimately found an absolute improvement in survival of 5% at five years in patients who received neoadjuvant platinum-based therapies compared to those who underwent resection alone [6]. Similarly, the *SWOG 9900* trial, published in 2010, showed a trend toward improved OS with neoadjuvant carboplatin–paclitaxel chemotherapy in patients with stage IB-IIIA disease, though these results did not reach statistical significance, as the trial was closed early, due to adjuvant chemotherapy becoming the standard of care while the trial was still in process [9].

More recently, the 2014 NSCLC Collaborative Group meta-analysis demonstrated a significant survival benefit with neoadjuvant chemotherapy, predominantly in stage IB-IIIA disease. Pooled analysis across the 15 studies included in this study demonstrated an HR for OS of 0.87 (95% CI, 0.78–0.96), suggestive of a 13% improvement in OS, as well as an increase in 5-year OS from 40% to 45% in patients who received neoadjuvant therapy compared to resection alone [10].

Alongside studies of neoadjuvant chemotherapy, studies examining the role of adjuvant therapy in NSCLC began to emerge in the 1990s. One early meta-analysis published in 1995, which included eight randomized trials, showed a small, but not statistically significant, benefit of approximately 5% at 5 years (*p* = 0.08) for patients who received adjuvant cisplatin-based therapy [11]. The International Adjuvant Lung Cancer Trial (*IALT*), published in 2004, expanded on these earlier studies and established systemic therapy as the standard of care in early-stage, resectable NSCLC. In this randomized control trial, patients who were randomized to 3–4 cycles of adjuvant cisplatin-based therapy saw a 5-year OS of 44.5%, compared to 40.4% in the control group, with an HR for death of 0.86 (95% CI, 0.76–0.98), indicative of a 14% reduction in the risk of death for patients receiving adjuvant therapy [12] The *JBR*.*10* trial, which specifically examined the role of the adjuvant in stage II disease, demonstrated substantial survival benefit, with an HR for death of 0.78 (95% CI, 0.61 to 0.99; *p* = 0.04) [13]. Like *JBR*.*10*, the Adjuvant Navelbine International Trialist Study (*ANITA*) likewise re-demonstrated the survival benefit of adjuvant therapy seen across all stages of resectable NSCLC in the *IALT*, specifically in patients with stage II and IIIA disease, with a significant improvement in OS from 37 to 51 months and with an HR for death of 0.79 (95% CI, 0.66–0.94) [14].

Pooled data from these studies on adjuvant therapy were compiled in the *LACE* meta-analysis, which showed an overall HR of death of 0.89 (95% CI 0.82–0.96, *p* = 0.005). The 5-year survival showed a 5.4% absolute improvement in the adjuvant therapy group, which improved from 60% to 65.4% [2]. However, this benefit was most pronounced in patients with stage II and IIIA disease, with a less-clear benefit observed in those with IB disease. *CALGB 9633* attempted to further explore the role of adjuvant chemotherapy in stage IB disease but failed to show a definitive, significant benefit overall. However, the results did trend toward increased benefit with a larger tumor size (≥4 cm), suggesting that tumor size may be an important factor in the use of adjuvant chemotherapy for patients with stage IB NSCLC [15].

While the extensive research described here has established a clear role for perioperative chemotherapy in the management of resectable NSCLC, the advent of the immune checkpoint inhibitor era in the past 10 years has further revolutionized the management of NSCLC. The immune checkpoint inhibitors available for clinical use primarily target the programmed death 1/program death 1 ligand (PD-1/PD-L1) and cytotoxic T-lymphocyte-associated protein 4 (CTLA-4) to reprogram the immune system that has been evaded by cancer [16].

There are now seven distinct immunotherapy agents approved as systemic therapy options in advanced/metastatic NSCLC: atezolizumab, cemiplimab, durvalumab, ipilimumab, nivolumab, pembrolizumab, and trememlimumab. More recent studies have investigated the benefit of immunotherapy in the treatment of resectable NSCLC, in both the adjuvant and neoadjuvant settings. Preclinical models have demonstrated improved antitumor activity with a neoadjuvant immunotherapy approach, owing to the presence of increased tumor antigens from unresected malignant lesions [16,17]. The current evidence for and the future directions of perioperative immune checkpoint therapy in resectable NSCLC will be discussed at length in the body of this review.

It is important to note here that the standard of care in the perioperative management of NSCLC is variable in the setting of certain molecular subpopulations, for which targetable therapies exist. Mutations for which targeted therapies are approved in the first-line setting treatment of advanced/metastatic NSCLC include *EGFR*, *EGFR ex20ins*, *MET14skip*, *BRAF V600E*, *ALK*, *ROS1*, *RET*, and *NTRK* [17]. The evidence for targeted therapies in these subpopulations highlights the importance of biomarker testing and adds to the complexity of treatment planning in these patients. Currently, two tyrosine kinase inhibitors—osimertinib in *EGFR* mutant and alectinib in *ALK*-rearranged NSCLC—have been approved by the FDA for treatment of this disease in the adjuvant setting. The role of perioperative targeted therapies will be further examined later in this review.

The literature was obtained through PubMed searches using the keywords perioperative non-small cell lung cancer, as well as index searches of major oncologic meetings, including the American Society of Clinical Oncology, the European Society for Medical Oncology, the World Conference on Lung Cancer, and the American Association for Cancer Research. With this introductory foundation, the authors in this narrative review discuss the current role and future perspectives of the use of immunotherapy and targeted therapy in the adjuvant and neo-adjuvant settings of patients with NSCLC who are deemed to have resectable disease. The management of the N2 resectable disease will also be discussed.

## 2. Determining the Need for Systemic Therapy in Operable NSCLC: Diagnosis, Staging, and Patient Performance Status

The paradigm for NSCLC staging, as set forth by the most recent edition of the American Joint Committee (AJCC) on Cancer in 2025, incorporates the T, N, and M categories to generate an overall disease stage, ranging from IA-IVB [18]. As with AJCC staging in other cancers, the T stage is dependent upon the primary tumor size and local invasion; the N stage upon the extent of local lymph node involvement; and the M stage upon the presence and degree of metastasis. While advanced imaging, such as CT and PET, has a role in determining disease staging, the gold standard of diagnosis remains tissue sampling, whether via IR-guided techniques, bronchoscopy with endobronchial ultrasound (EBUS), or video-assisted thoracic surgery (VATS) [19]. Tissue sampling is of special importance not only for the establishment of general diagnosis but for the purpose of molecular testing, which guides therapeutic decisions based on the presence of targetable mutations and PDL1 expression [16,19].

Generally speaking, patients with a stage I–IIIA disease burden are considered surgical candidates. However, the ultimate determination of operability is largely driven by individual thoracic surgery evaluation and multidisciplinary tumor board discussions [20].

While the prior studies discussed at length above have clearly established a role for chemotherapy in the management of operable NSCLC, it is important to note that perioperative chemotherapy is not necessarily appropriate in all patients. At this juncture, based on the evidence established in prior studies investigating the impact and feasibility of perioperative chemotherapy, specifically in the *CALGB* study, as alluded to above, the size of tumor, presence of lymph node metastasis, and baseline patient performance status are the most important factors in determining the role for perioperative therapy [3,15]. In the case of patients with stage IB disease, for instance, there is questionable benefit from perioperative treatment if the primary tumor size is <4 cm, based on the *CALGB* trial [15].

Furthermore, patient functional status also plays a significant role in the expected benefit of perioperative chemotherapy. The ECOG scale (Eastern Cooperative Oncology Group) is a well-established, standard method of ranking patient fitness, with scores ranging between zero (fully active, able to carry on all pre-disease activities) and five. In patients with an ECOG score of two or greater, which correlates to the ability to carry out all self-care activities but restricted from any work-related activities, perioperative chemotherapy is less likely to be associated with the same survival benefit seen in patients with a better baseline functional status [21,22]. This lack of benefit from chemotherapy in such patients is suspected to result from a combination of decreased baseline physiologic reserve, diminished efficacy of systemic therapy, increased toxicity risk resulting in more frequent dose reductions or treatment discontinuation, poor quality of life, and shorter survival time related to other underlying comorbidities [23].

In sum, based on these factors, the decision to initiate perioperative treatment becomes even more important and should be individualized based on specific patient characteristics. As such, the studies discussed in this review cannot and should not be blindly applied to all patients diagnosed with operable NSCLC without a more nuanced analysis of individual considerations.

## 3. Surrogate Endpoints for Overall Survival in Adjuvant and Neoadjuvant/Perioperative Trials for NSCLC

In clinical trials for neoadjuvant and adjuvant therapies in NSCLC, overall survival (OS) remains the gold-standard endpoint to measure the efficacy of treatments. However, due to the long follow-up periods needed to observe survival differences, surrogate endpoints are often used to provide earlier indications of clinical benefit. First described by RL Prentice in 1989, surrogate endpoints are defined by three distinct rules: the treatment intervention must be associated with the surrogate, the surrogate must be associated with the true outcome, and the surrogate must be able to explain the entirety of the effect on the true outcome [24]. Starting with the approval of IL-2 for metastatic renal cell cancer in 1992, the FDA has accepted surrogates that are reasonably likely to be associated with clinical benefit and has approved 194 oncologic drugs between 1992 and 2019, representing about 33% of all approvals [25].

Disease-free survival (DFS) refers to the time from randomization to the first event, namely locoregional recurrence, distant recurrence, or death from any cause. DFS is a frequently used surrogate for OS in adjuvant trials and validated in meta-analysis of over 7500 NSCLC patients treated with adjuvant chemotherapy and a correlation coefficient ranging from R^2^ = 0.92 (95% CI 0.88–0.92) to R^2^ = 0.99 (0.98–1.00) depending on the addition of radiation [26]. While generally translating to better outcomes, the use of DFS as a surrogate endpoint is limited by the effect of later lines of treatment, which may prolong survival despite disease recurrence.

Event-free survival (EFS) is similar to DFS but includes any disease progression or death prior to surgery in neoadjuvant trials. It can also include failure to undergo surgery as an event. EFS is commonly used in neoadjuvant settings because it captures not only cancer recurrence but also the impact of neoadjuvant therapy on surgical outcomes. If neoadjuvant therapy increases the likelihood of complete resection, this would likely be reflected in improved EFS. Its role as a surrogate marker in resectable NSCLC was evaluated in a meta-analysis by Ostoros et al., showing a Pearson’s correlation coefficient of r = 0.864 (95% CI 0.809–0.992) [27]. However, like DFS, EFS may not perfectly correlate with OS, especially in patients who can be salvaged after recurrence with additional treatment.

Pathologic complete response (pCR) refers to the absence of any residual viable tumor cells in the resected lung tissue and lymph nodes following neoadjuvant therapy. The pCR is widely used as a surrogate in neoadjuvant trials, particularly with immunotherapies and targeted therapies. Achieving pCR is often associated with a lower risk of recurrence and better long-term survival, although the strength of this correlation can vary across studies [28,29,30]. However, not all patients who achieve pCR will experience long-term survival, and conversely, some patients without pCR may still do well. It is a strong surrogate, especially given its early readout, but it is not always a perfect predictor of OS.

Major pathologic response (MPR) is defined as ≤10% of residual viable tumor cells in the primary tumor following neoadjuvant therapy. MPR is gaining popularity as a surrogate endpoint in neoadjuvant trials. Like pCR, it serves as an early indicator of how effective the neoadjuvant treatment has been. MPR correlates well with long-term outcomes, and some studies suggest it might be a more sensitive marker of treatment efficacy compared to pCR [31,32]. However, while MPR is predictive of OS in some studies, the relationship is not universal, and not all patients who achieve MPR will have improved survival.

Recurrence-free survival (RFS) is the time from treatment to the first occurrence of cancer recurrence (local, regional, or distant) but does not consider death from other causes. RFS can be an appropriate endpoint in early-stage NSCLC patients where death from other causes might confound a survival analysis, especially in older populations or for accelerated FDA approval [33]. RFS does not account for death from non-cancer causes, and this can limit its utility as a direct surrogate for OS in certain populations.

Progression-free survival (PFS) is the time from the start of treatment (neoadjuvant or adjuvant) until the disease progresses (locally or distantly) or the patient dies from any cause. PFS is frequently used in both neoadjuvant and adjuvant trials, especially when studying therapies aimed at delaying the progression of the disease. While PFS provides an early signal of treatment efficacy, it may not always correlate well with OS, particularly in settings where effective subsequent treatments are available after progression [33].

Minimal residual disease (MRD) refers to the detection of small numbers of cancer cells that remain in the body after treatment and are typically identified using molecular or circulating tumor DNA (ctDNA) assays. Emerging as a novel surrogate endpoint, MRD negativity after neoadjuvant or adjuvant treatment is associated with better outcomes, as it suggests that the treatment has eradicated nearly all tumor cells [34,35,36]. The utility of MRD as a surrogate is still being validated in NSCLC and is discussed in greater detail in this review.

Time to recurrence (TTR) is the time from surgery to the recurrence of NSCLC. It is similar to DFS, but it excludes death from other causes as an event. TTR can help isolate the effects of treatment on tumor biology without being confounded by unrelated deaths. Like other recurrence-based surrogates, it may not perfectly predict OS, especially in older populations where competing causes of mortality are common [37].

In conclusion, surrogate endpoints like DFS, EFS, pCR, MPR, and MRD are invaluable tools in neoadjuvant and adjuvant NSCLC trials, allowing for earlier assessments of treatment efficacy. However, their correlation with overall survival must be critically evaluated in each trial to ensure that these endpoints are meaningful in the long term (Table 1).

## 4. Adjuvant and Neoadjuvant/Perioperative Trials in NSCLC Utilizing Checkpoint Inhibition

Within the last 7 years, a series of trials investigating the role of perioperative immunotherapy in resectable NSCLC have been published. In this section, the authors highlight the study design and primary results of each, starting with early-phase immunotherapy trials, followed by chemoimmunotherapy, phase III landmark studies, the use of targeted therapies, and lastly, future directions.

### 4.1. Overview of Phase I–II Trials Investigating Neoadjuvant Immunotherapy in NSCLC Management

The beginning of perioperative immunotherapy investigation was marked in 2018 with the publication of the pilot study of Neoadjuvant PD-1 Blockade in Resectable Lung Cancer. Twenty-one patients with surgically resectable stage I–IIIA NSCLC at Johns Hopkins Sidney Kimmel Comprehensive Cancer Center and Memorial Sloan Kettering Cancer Center were enrolled to receive two doses of nivolumab (at 3 mg/kg) over 4 weeks prior to surgical resection. The study demonstrated an MPR rate of 45%, with two patients achieving a pCR, and 20 of the 21 enrolled patients underwent complete resection with an 18-month DFS rate of 73%. This intervention was well-tolerated, with only one grade 3 or higher toxicity [38].

This was subsequently followed in 2021 by the publication of the *NEOMUN* trial. The first study utilizing the PD-1 inhibitor pembrolizumab in the neoadjuvant space enrolled 15 stage II/IIIA patients to receive a fixed dose of 200 mg of pembrolizumab every 3 weeks for two cycles prior to surgery. Twelve of 15 patients received two doses of pembrolizumab without deviation—two developed a grade 3 treatment-related adverse event (TRAE), and another required a delay to complete steroid for a grade 2 AE. All patients underwent an R0 resection, and 27% achieved an MPR with two pCRs. Notably, this study also demonstrated that metabolic tumor target response, defined as > than a 25% decrease in SUV_max_, correlated with a significantly higher MPR/pCR rate (*p* = 0.015). Additionally, PD-L1 expression greater than 10% was also correlated with a higher MPR/pCR rate (*p* = 0.033) [39].

The open-label single-arm phase II *LCMC3* trial enrolled 181 stage IB-IIIB patients without *EGFR* or *ALK* alterations to receive two doses of PD-L1 inhibitor atezolizumab at 1200 mg every 21 days prior to surgery. Of the 143 patients who underwent surgery and were included in primary end-point analysis, 20% achieved an MPR (95% CI 14–28%), correlating with a 3-year OS rate of 80%. Interestingly, when stratified by PD-L1 TPS < 1%, 1–49%, and >50%, the MPR rates were 11% (6/53), 5% (1/20), and 33% (15/45), respectively. LCMC3 included an exploratory analysis that evaluated the peripheral blood gene expression of NK cells compared to RNA sequencing of the tumor microenvironment NK cells to predict MPR, finding that coexpressions of immunoglobulin-like \transcript 2 (ILT-2) and PD-L1 were associated with MPR in nonsquamous histology [40].

The addition of CTLA-4 inhibitor to PD-1 blockade was examined in the phase II *NEOSTAR* trial; 44 patients with stage I–IIIA NSCLC were randomized at a 1:1 ratio to three doses of nivolumab dosed at 3 mg/kg every two weeks with or without the addition of the CTLA-4 inhibitor ipilimumab 1 mg/kg on day 1. The primary endpoint of the MPR was reached in 22% of the nivolumab arm (5/23), compared to 38% of the dual checkpoint arm (8/21). When compared to nivolumab, the addition of ipilimumab also resulted in a higher pCR rate (38% vs. 10%), a less viable tumor (median 9% vs. 50%), and a similar grade 3–5 treatment-related AE rate (10 vs. 13%). An analysis of the pretreatment PD-L1 IHC demonstrated a 33% MPR/pCR rate for PD-L1 non-expressors receiving dual checkpoint inhibition (3/9) compared to 0% for nivolumab alone (0/7), suggesting an increased benefit in dual checkpoint inhibition in this subpopulation [41].

### 4.2. Phase II Trials Examining Perioperative Chemoimmunotherapy in NSCLC

The combination of neoadjuvant immunotherapy with chemotherapy was first investigated in the investigator-initiated, phase II trial of neoadjuvant atezolizumab and chemotherapy trial published by Shu et al. in 2020 [42]. Thirty patients with stage IB-IIIA received two cycles of 1200 mg atezolizumab and carboplatin [area under the curve (AUC) 5] on day 1 and nab-paclitaxel (100 mg/m^2^) on days 1, 8, and 15. Nab-paclitaxel was chosen specifically as part of the chemotherapy doublet to avoid steroid premedication, which diminishes immunotherapy response. Patients without CT confirmed radiographic progression after two cycles proceeded, with two additional cycles followed by surgical resection. Twenty-nine patients proceeded with surgical resection, of which 17 (57%, 95% CI 37–75) obtained an MPR, 10 (33%, 95% CI 17–53) obtained a pCR and 11/19 (58%) downstaged from N2 to N0 as a result of neoadjuvant chemoimmunotherapy. Treatment was generally tolerable, with three serious TRAEs and no treatment-related surgical delays [42].

The single-arm, open-label phase II *NADIM* trial, originally published in 2020, was the first to utilize neoadjuvant chemoimmunotherapy followed by adjuvant immunotherapy. In this study, 46 patients with stage IIIA NSCLC received three cycles of paclitaxel (200 mg/m^2^), carboplatin (AUC 6), and nivolumab (360 mg) followed by surgical resection and 1 year of adjuvant nivolumab monotherapy. A recently published 5-year follow-up showed a PFS rate of 65% (95% CI 49.4–76.9) and an OS of 69.3 (95% CI 53.7–80.6). Secondary endpoints include an MPR rate of 84% and a pCR of 63%, both significantly higher than historical controls [43,44].

Given the success of *NADIM*, a follow-up phase II *NADIM II* was designed. It assigned 86 patients with IIIA or IIIB in a 2:1 fashion to neoadjuvant chemoimmunotherapy, as per NADIM, or neoadjuvant chemotherapy with three cycles of carboplatin (AUC 5) and paclitaxel (200 mg/m^2^). Those in the experimental arm who underwent R0 resection proceeded with 6 months of nivolumab. The pCR rate in the experimental arm was 37% compared to 7% in the control arm, conferring a relative risk of 5.34 (95% CI 1.34–21.23). The 24-month PFS rate was 67.2% compared to 40.9% in the control arm, and the OS at 24 months was also improved with the addition of immunotherapy, with a hazard ratio of 0.43 (95% CI 0.19–0.98). Given the clinical success of the combination of chemotherapy with immune therapy, coupled with a manageable safety profile, these regimens were moved to the phase III setting [43].

### 4.3. Overview of Phase III Trials Investigating Neoadjuvant/Perioperative Immunotherapy in NSCLC Management

Published in 2022, the *Checkmate 816* trial represents one of the first large investigations into the benefit of immunotherapy in resectable NSCLC, focusing strictly on the role of neoadjuvant chemoimmunotherapy prior to surgical resection. The study consisted of an open-label, randomized, phase III trial in which patients with resectable NSCLC were randomly assigned to receive either three cycles of neoadjuvant nivolumab plus platinum-based chemotherapy or three cycles of neoadjuvant chemotherapy alone, followed by surgical resection. The trial enrolled a total of 358 patients, who were followed for a minimum of 21 months. The enrollment criteria included diagnosis of stage IB (≥4 cm) to IIIA (AJCC 7th Edition) NSCLC, ECOG status of 0–1, no prior anticancer therapy, and pre-treatment tissue available for analysis of PDL1 expression. Patients with *ALK* translocations or *EGFR* mutations were excluded. Primary endpoints included event-free survival and pathological complete response, with the overall survival (OS) as a secondary endpoint. 

Upon analysis, the trial cohort demonstrated a median EFS of 31.6 months (95% CI 30.2 months—time not reached in follow-up) vs. 20.8 months (95% CI 14–26.7) in those receiving standard chemotherapy alone (HR 0.63, 97.38% CI 0.43–0.91, *p* = 0.005). Likewise, combination neoadjuvant nivolumab and platinum-based chemo showed a statistically significant advantage in terms of pCR in comparison to neoadjuvant chemotherapy alone (24%, 95% CI 18–31 in patients receiving immunotherapy vs. 2.2, with 95% CI 0.6–5.6 in patients receiving chemotherapy alone and an OR = 13.94 [3.49–55.75], *p* < 0.001). The most recently presented 57.6 months median follow-up data continues to demonstrate a favorable median EFS (43.8 months vs. 18.4 months; HR [95% CI], 0.66 [0.49–0.90]), with 49% of the patients who received chemoimmunotherapy alive and free of disease recurrence at 4 years, compared to 38% of the patients who received chemotherapy alone. The median OS has not yet been reached in both arms, but trends towards superiority for the chemoimmunotherapy group (4-year OS at 71% vs. 58%, HR [98.36% CI], 0.71 [0.47–1.07]; *p* = 0.0451) [45]. Notably, however, patients who achieved a pCR had an improved OS compared to those who did not, with a 4-year OS rate of 95% compared to 63% [HR 0.08, (95% CI 0.02–0.34)]. Furthermore, the addition of neoadjuvant nivolumab did not result in an increase in overall treatment-related adverse events, with adverse events observed in 92.6% of patients receiving experimental treatment vs. 97.2% of patients receiving chemotherapy alone. No treatment-related deaths were observed in the nivolumab group, compared to three deaths in the chemotherapy-only group [46].

Building on the *Checkmate 816* trial, *Checkmate 77T* also studies the efficacy of perioperative chemoimmunotherapy, designed to include both neoadjuvant and adjuvant nivolumab. *Checkmate 77T* randomized a total of 461 patients with AJCC 8th Edition Classification stage IIA-IIIB (N2) NSCLC to receive either four cycles of neoadjuvant platinum-based chemotherapy plus nivolumab, followed by adjuvant nivolumab every four weeks for one year, or four cycles of chemotherapy plus placebo, followed by one year of placebo. The primary outcome was EFS, with pCR, MPR, OS, and safety as secondary outcomes. As in *CheckMate 816*, patients with *EGFR* mutations and known *ALK* translocations were excluded.

At the prespecified 18-month interim analysis mark, a statistically significant increase in EFS in patients receiving perioperative nivolumab was noted, with an EFS rate of 70.2% in the nivolumab group compared to 50% in the placebo group (HR 0.58, 95% CI 0.42–0.81, *p* < 0.001). Likewise, a pathological complete response was achieved in 25.3% of patients in the immunotherapy arm, compared to only 4.7% in the chemotherapy group (OR 6.64, 95% CI 3.40–12.97).

By comparison, patients receiving neoadjuvant nivolumab alone in the *Checkmate 816* trial exhibited a 24% pCR rate. By extrapolation of the Kaplan–Meier curve from the *Checkmate 816* trial, event-free survival at 18 months for patients receiving neoadjuvant nivolumab falls between 63.8 and 76.1%. While it is challenging to compare EFS across these two studies, given geographic and patient variation, it raises an interesting point as to the added benefit of an additional cycle of chemoimmunotherapy, as well as that of combined neoadjuvant/adjuvant nivolumab compared to neoadjuvant therapy alone. This variation in post-operative standard of care represents an important challenge in determining subpopulations who benefit from additional therapy following surgical resection and will be discussed at further length in this review [47].

*Keynote-671* was designed as a randomized, double-blinded phase III trial investigating the utility of perioperative pembrolizumab in patients with resectable NSCLC. The trial enrolled a total of 797 patients with resectable AJCC 8th Edition Classification stage II, IIIA, and IIIB N2 disease, who received either neoadjuvant pembrolizumab or placebo once every 3 weeks with cisplatin-based chemotherapy for 4 cycles, followed by surgery and adjuvant pembrolizumab or placebo once every 3 weeks for up to 13 cycles. Similar to *Checkmate 77T*, the inclusion criteria included an ECOG score of 0–1 and pre-treatment tissue available for analysis of PDL1 expression. The primary endpoints included OS and EFS, with pathologic response by central examination and safety as secondary endpoints. 

A total of 397 participants were randomized to the pembrolizumab group and 400 to the placebo group, with a median follow-up time of 25.2 months. Data analysis revealed a statistically significant difference in EFS, with patients in the pembrolizumab arm exhibiting 62.4%, compared to 40.6% in the control group (HR 0.58, 95% CI 0.46–0.72, *p* < 0.001). Further, a pCR was observed in 18.1% of cases in the pembrolizumab arm, compared to 4.0% in the placebo arm (*p* < 0.0001). As with previously described perioperative chemoimmunotherapy trials, the OS data are still immature, with an 80.9% OS rate in the pembrolizumab group vs. 77.6% in the placebo group (*p* = 0.02). There was no statistically significant difference in the total TRAEs in the experimental group (96.7% in the pembrolizumab group, 95% in the placebo group) or in the number of treatment-related deaths (four in the pembrolizumab group, three in the placebo group). A total of 33 patients with known *EGFR* mutations were randomized, and an HR of 0.09 for DFS (95% CI 0.01–0.074) was reported. However, in view of the very small number of patients and the extensive prior data demonstrating a lack of benefit of checkpoint inhibitors in patients with EGFR mutations, this result should be interpreted with extreme caution and is not sufficient to support the use of perioperative pembrolizumab for such patients. The most commonly reported adverse effects in both groups were nausea, decreased neutrophil count, anemia, leukopenia, and fatigue [48].

Similarly to *CheckMate 77T* and *Keynote-671*, the *AEGEAN* trial investigated the efficacy of a combined neoadjuvant and adjuvant course of immunotherapy compared to standard chemotherapy, with the PD-L1 inhibitor durvalumab used as the experimental drug. In this randomized control trial, a total of 802 participants with 8th Edition AJCC classification stage IIA to stage IIIB (N2) NSCLC were randomized to receive either four cycles of neoadjuvant platinum-based chemotherapy plus durvalumab, followed by adjuvant durvalumab every four weeks for 12 cycles, vs. four cycles of neoadjuvant platinum-based chemotherapy with placebo, followed by 12 cycles of placebo adjuvant therapy. Data from the 62 patients with documented *EGFR* or *ALK* alterations were excluded from analysis. The inclusion criteria in the study closely matched those utilized *in Checkmate 77T* and *Keynote-671*, namely an ECOG of zero or one and documentation of PDL1 expression.

In keeping with other neoadjuvant/perioperative trials of chemoimmunotherapy, the *AEGEAN* study demonstrated a statistically significant difference in pCR between the treatment and placebo groups, which was observed in 17.2% of the patients receiving durvalumab vs. 4.3% in those receiving chemotherapy alone (95% CI, 8.7 to 17.6; *p* < 0.001). A statistically significant difference in EFS was observed for the durvalumab-containing arm, with 73.4% vs. 64.5% and 63.3% vs. 52.4% at 12 and 24 months, respectively, HR 0.68 (95% CI, 0.53–0.88, *p* = 0.004). This benefit in event-free survival was observed regardless of PD-L1 expression on subgroup analysis, though the magnitude of benefit seemed greater in patients with PD-L1 > 50%, HR 0.60 (95% CI 0.33–1.38). 

A review of safety data from the trial reveals a similar rate of adverse events in patients receiving durvalumab compared to chemotherapy alone, with an overall AE rate of 96.5% in the durvalumab group compared to 94.7% in the chemotherapy group. While immune-related adverse effects were unsurprisingly more common in the durvalumab group (23.7% vs. 9.4%), most were grade 1 or 2. Only seven patients had surgery cancelled in the durvalumab-containing arm, compared to four patients in the placebo group [49].

Toripalimab, a PD-1 inhibitor FDA approved for advanced nasopharyngeal carcinoma, has also been examined in the perioperative space in the *Neotorch* trial, which published an interim analysis in 2024. *Neotorch* randomized 404 patients with stage III NSCLC in a 1:1 ratio to receive three cycles of platinum doublet chemotherapy with or without 240 mg toripalimab, followed by surgical resection, and one subsequent cycle of adjuvant chemotherapy with either 1 year of adjuvant toripalimab or a placebo. The coprimary outcomes were investigator-assessed event-free survival and MPR rate, with the secondary outcomes including pCR rate and safety. The median EFS rate in the toripalimab cohort was not reached (95% CI, 24.4 months-not estimable) compared with an EFS rate of 15.1 months (95% CI, 10.6–21.9 months) in the placebo group [HR 0.40, (95% CI, 0.28–0.57), *p* < 0.001]. Evaluation of the second coprimary endpoint major pathological response revealed a 48.5% MPR rate (95% CI, 41.4–55.6%) in the toripalimab group compared with an 8.4% (95% CI, 5.0–13.1%) in the placebo group, indicating a between-group difference of 40.2% [95% CI, 32.2–48.1%], *p* < 0.001). An analysis of the secondary endpoint pathological complete response demonstrated a 23.7% increase in the experimental arm, with a pCR rate of 24.8% (95% CI, 19.0–31.3%) in the toripalimab group compared to 1.0% (95% CI, 0.1–3.5%) in the placebo group. Toripalimab was generally well-tolerated, with a grade III or higher TRAE rate of 63.4% in the experimental arm compared to 54% in the placebo, and one death related to checkpoint inhibitor pneumonitis.

While *Neotorch* provides additional evidence of the efficacy of perioperative chemoimmunotherapy, this trial enrolled 77.7% of patients of squamous histology, and 90% of the patients enrolled were of male sex, not reflecting the demographics seen in the United States. Toripalimab is now approved in China for this indication [50] (Table 2).

## 5. Phase III Trials of Adjuvant Immunotherapy in Resected NSCLC

The use of adjuvant chemotherapy for resected (IB-IIIA) NSCLC has had a defined role for close to two decades, with a *LACE* meta-analysis showing that the addition of adjuvant cisplatin doublet chemotherapy provides a modest 5.4% survival benefit, which is improved from 60% to 65.4% [2]. This benefit was most pronounced in patients with stage II and III, and very importantly, patients with borderline functional status ECOG 2 enrolled in such trials fared best in the control arms. Naturally, with the advent of checkpoint inhibition in the advanced/metastatic setting, studies evaluating its effect in the post-operative phase followed suit.

The first phase III trial evaluating the use of adjuvant PD-L1 blockade was published in 2021. *IMpower010* randomized 1005 stage IB (≥4 cm) to IIIA NSCLC patients (per AJCC 7th Edition) in a 1:1 manner after completed resection and adjuvant cisplatin doublet chemotherapy to receive 1200 mg atezolizumab every 21 days for 16 cycles or best supportive care (BSC). In addition to the large study size, *IMpower010* is also notable for the inclusion of patients with known *EGFR*/*ALK* alterations and evaluation by a higher-stage, PD-L1 positive subpopulation (II–IIIA) and in the intention-to-treat (ITT) population encompassing all patients. The primary outcome was DFS in all populations, with a key secondary outcome, DFS in the stage II–IIIA PD-L1 high expressing (>50%) subpopulation, but also evaluated in all patients with stage II–IIIA disease and in the ITT population. Secondary endpoints included overall survival in the ITT population, DFS in stage II–IIIA PD-L1 ≥ 50%, 3- and 5-year DFS in stage II–IIIA PD-L1 ≥ 1%, all stage II–IIIA patients and finally the whole ITT population.

The results of *IMpower010* were mixed. In the ITT population encompassing all patients stage IB-IIIA, the 3-year DFS rates were 57.9% in the atezolizumab arm and 52.6% in the BSC arm, with a stratified HR of 0.81 (95% CI 0.67–0.99, *p* = 0.04). In the population of all patients who are stage II–IIIA, the 3-year DFS rates were 55.7% in the atezolizumab arm and 49.4% in the BSC arm, with a stratified HR of 0.79 (95% CI 0.64–0.96, *p* = 0.02). While both met clinical significance to the primary endpoint, there was an 18% serious AE rate in the atezolizumab arm compared to 8% in the BSC arm, suggesting the importance of counseling patients in these cohorts.

However, in the stage II–IIIA PD-L1 expressor population, the median DFS was not reached (95% CI 36.1 months—not reached) in the atezolizumab arm compared to 35.3 months (95% CI 29.0—not reached) in the BSC arm, with a stratified HR of 0.66 (95% CI 0.5–0.88, *p* = 0.0039). In this same population, the 3-year DFS rates were 60% in the experimental arm compared to 48.2% in the BSC arm. Further subgroup analysis noted that the stage II–IIIA population with a PD-L1 expression of 50% showed an unstratified HR of 0.43 (95% CI 0.27–0.68), a key secondary endpoint, suggesting that much of the benefit from atezolizumab is seen in patients harboring tumors with high PD-L1 expression. Given the results of *IMpower010*, the role for adjuvant PD-L1 blockade after chemotherapy in patients who are stage II–IIIA with a PD-L1 expression was established [51,52]. While FDA-approved for adjuvant chemotherapy in patients with stage II–III NSCLC with PD-L1 ≥ 1%, the European Medicines Agency requires high PD-L1 levels (≥50%) for patients to qualify.

Following *IMpower010*, the *PEARLS*/*Keynote-091* investigators published an interim analysis in 2022 examining the role of adjuvant PD-1 blockade with pembrolizumab. *PEARLS*/*Keynote-091* randomized 1177 patients at 196 sites with completely resected stage IB (≥4 cm)-IIIA NSCLC (AJCC 7th Edition) to receive either pembrolizumab 200 mg or placebo every 3 weeks for up to a year. Patients were to be “considered” and “strongly recommended” to receive adjuvant chemotherapy for stage IB and stage II–IIIA disease, respectively. The dual primary endpoints were DFS in the ITT population and the PD-L1 TPS high (≥50%) subgroup. The secondary endpoints in *PEARLS*/*Keynote-091* included OS in the ITT, high-expressing PD-L1 and PD-L1 ≥ 1% populations, and DFS in the PD-L1 positive strata. Similar to Impower 010, patients with known *EGFR*/*ALK*-positive tumors could enroll and accounted for 7.3% of the randomized patients.

The study achieved its first coprimary endpoint of median DFS in the ITT population at 53.6 months (95% CI 39.2—not reached) in the pembrolizumab arm compared to 42.0 months (95% CI 31.3- not reached) in the placebo arm, sufficient for an HR of 0.76 (95% CI 0.63–0.91, *p* = 0.0014). Interestingly however, there was no significant DFS benefit for the PD-L1 high expression group, where the median DFS was not reached in either the pembrolizumab arm (95% CI 44.3—not reached) nor the placebo arm (95% CI 35.8—not reached), correlating with a HR of 0.82 (95% CI 0.57–1.18, *p* = 0.14). This was certainly unexpected and diverges from the abundance of data gathered in the advanced/metastatic setting, where patients with the highest PD-L1 expression consistently demonstrate the best outcomes with pembrolizumab. The reasons for this finding remain unclear to date, but it is speculated that it may be attributed in part to the favorable outcomes of the control arm. Non-recipients of adjuvant chemotherapy did not benefit from adjuvant pembrolizumab (HR 1.25; 95% CI 0.76–2.05). Overall survival data remains immature to date. Given the DFS benefit in the ITT population, pembrolizumab carries an FDA indication for stage IB-IIIA, regardless of PD-L1 status [53].

While *IMpower010* and *PEARLS*/*Keynote-091* established the current treatment paradigm of adjuvant immunotherapy after resected NSCLC, there are other trials yet to report out that are forthcoming. *ANVIL* (EA5142), an *ALCHEMIST* trial investigating the use of PD-1 blockade with nivolumab in adjuvant stage IB (≥4 cm)-IIIA NSCLC, has completed enrollment [54]. It is important to note that all these trials are sequenced after resection and chemotherapy rather than in lieu of chemotherapy, which still has a defined role in the adjuvant treatment of NSCLC. Moreover, patients with the borderline performance status ECOG 2 were not enrolled in *PEARLS*/*KEYNOTE-091* and accounted for less than 1% of the patients in *IMpower 010*.

## 6. Examining the Role of Targeted Therapy in Perioperative NSCLC

The role for perioperative therapy becomes even more nuanced in the case of patients with NSCLC with certain actionable genomic alterations. While chemoimmunotherapy establishes its role in the treatment of operable NSCLC, the same cannot be said for the subgroups with *EGFR*/*ALK*-positive tumors. Multiple studies in the advanced/metastatic setting have consistently demonstrated minimal or no benefit of checkpoint inhibitor therapy, and most trials in the perioperative space have excluded such patients or enrolled at minimal capacity [55,56]. As a result, research regarding the role of targeted therapies in the perioperative setting of resectable NSCLC has only more recently come under investigation. Impactful results of the TKI-adjuvant trials in relation to *EGFR* mutant and *ALK*-rearranged NSCLC have led to the FDA approvals of osimertinib and alectinib for surgically treated disease and affirmed the need for the genomic testing of patients with NSCLC at all levels of care [57,58]. We look forward to the output of many emerging clinical trials that examine the role of targeted therapies in other proto-oncogene mutations (NCT03157128, NCT05472623, NCT04302025, NCT05118854) [59,60,61,62].

### 6.1. Phase II Trials in Neoadjuvant and Perioperative Targeted Therapy for NSCLC 

One of the earliest trials examining the role of neoadjuvant and perioperative TKI therapy in patients with *EGFR* + NSCLC was published in 2009, demonstrating a positive signal for gefitinib, with an ORR of 11% amongst the 36 study participants in this single-arm study [63].

A small study in China with an interesting design enrolled 24 patients with stage IIIA-N2 NSCLC to receive pre-operatively either erlotinib or gemcitabine/carboplatin (GC) based on genomic testing results (*EGFR* mutant + versus *EGFR* wild type, respectively) [64]. The study aimed to compare a genomically-driven neoadjuvant approach with a standard of care cytotoxic chemotherapy in these two different patient groups, with the primary endpoint consisting of ORR and secondary endpoints including PFS and OS. Twelve of the patients enrolled (50%) had an *EGFR*m + tumor. Despite a numerically superior ORR (58.3% versus 25%, *p* = 0.18), a trend toward inferior PFS (6.9 months vs. 9.0 months, *p* = 0.071) and OS (14.5 months vs. 28.1 months) was demonstrated for the erlotinib-treated *EGFR*m + group compared to the GC-treated patients. Pathological CR in N2 stations was seen in 16.7% and 25% of the erlotinib and GC patients, respectively. These hypothesis-generating findings highlight the biological differences of tumors with AGAs versus non-AGA NSCLC, as well as how the mechanistically different treatment approaches (cytostatic versus cytotoxic therapy) may potentially impact outcomes in neoadjuvant settings.

A later 2021 phase II study of similar size by Zhang et al. further demonstrated 54.5% ORR and 24.2% MPR amongst study participants who received gefitinib in the neoadjuvant setting [65]. Additional single-arm studies, such as the ML25444 trial, further showed activity of neoadjuvant erlotinib in patients with *EGFR* mutations [66,67].

The *EMERGING-CTONG1103* trial built upon these early findings to examine the benefits of perioperative erlotinib vs. standard platinum-based chemotherapy in patients with stage IIIA-N2, *EGFR* mutant + disease. The patients were randomized to receive either 42 days of neoadjuvant erlotinib followed by 12 months of adjuvant TKI or four cycles of gemcitabine/cisplatin (GC) (two cycles delivered prior to and two cycles given after surgery). In this phase II trial, with an ITT number of 72 patients in China, an initial analysis showed a PFS of 21.5 months in the erlotinib group, compared to 11.4 months in the standard chemotherapy group, with a statistically significant HR of 0.39 (95% CI, 0.23–0.67) [68]. However, only a numerically small median OS difference between the two groups was demonstrated (42.2 in the erlotinib group vs. 36.9 months in the control group), failing to reach statistical significance [HR 0.83; (95% CI, 0.47–1.47) *p* = 0.513]. This could be explained in part due to the fact that patients in the control arm with recurrent disease were then exposed to the then-available TKI therapy [69].

A phase 2 trial looking at the efficacy and safety of 8–12 weeks of afatinib prior to surgical resection in patients with *EGFR* mutant + NSCLC enrolled 47 participants with stage IIIA-C disease. Despite an ORR of 70.2%, only 9.1% and 3% of the individuals achieved an MPR or pCR, respectively. Dynamic changes in the tumor microenvironment associated with response to afatinib were indicative of a pro-inflammatory response, with increased numbers of cytotoxic T-cells, B-cells, and NK-cells [70].

A few single phase II trials have been undertaken to investigate the benefits of neoadjuvant osimertinib. The 2021 phase 2b *NEOS* trial conducted in China demonstrated the clinical activity of neoadjuvant osimertinib in 40 patients with stage IIA-IIIB disease, with an ORR of 71.1% in 38 patients who completed 6 weeks of therapy prior to surgical resection [71]. The MPR rate in the 28 patients with evaluable pathology was 10.7%, and one patient achieved a pCR (3.6%). A subsequent phase II study conducted in the US by Blakely et al. demonstrated a DFS of 32 months and an MPR of 15% amongst the 27 recruited patients who received neoadjuvant osimertinib [72]. Though an ORR of 52% was observed, the MPR rate in the ITT population was only 14.8%. It did not meet the primary endpoint of 50%, and there were no pCRs. The authors explored the role of co-mutations in hampering the activity of directed TKI therapy in such patients and identified *RBM*_10_ LOF mutations in 15% of the patients, with none of such individuals achieving an MPR. However, this cannot explain the disparity between the MPR rates seen in trials such as this and neoadjuvant chemo-immunotherapy studies.

There are a few hypotheses that may explain the recurrent finding of relatively low rates of MPR and pCR in neoadjuvant trials using TKI therapy in patients with actionable genomic alterations. Unlike cytotoxic chemotherapy, TKIs are cytostatic in nature, primarily inhibiting cell proliferation rather than inducing direct cell death. Therefore, despite very high ORRs seen in imaging assessment, much lower MPR and pCR rates are documented. Another possibility is that the average duration of neoadjuvant TKI utilized in such trials (approximately 6 to 8 weeks) is too short for a greater extent of benefits to be observed with TKI therapy. Moreover, cancers harboring genomic alterations, such as *EGFR* and *ALK*, are typically non-immunogenic, and different from chemotherapy and immunotherapy, TKI therapy does not induce robust immune responses in relation to direct tumor cell death.

As it relates to *ALK-*rearranged NSCLC, there are a few studies examining the benefit of neoadjuvant/perioperative crizotinib, ceritinib, and alectinib in resectable, *ALK-*rearranged NSCLC. One study by Zhang et al., a single-arm study with 11 participants with N2 *ALK-*rearranged NSCLC, found a 90.9% ORR in patients receiving neoadjuvant crizotinib for a median duration of 30 days (range 28–120), and two patients (18.2%) achieved a pCR [73]. The median time between the patients’ last crizotinib dose and surgery was 11 days. In a single institutional retrospective study by the same group, 39 consecutive patients with pathologically confirmed stage IIIA-B *ALK-*rearranged NSCLC and treated with either neoadjuvant crizotinib or alectinib were evaluated. Only 74.3% of the patients underwent surgery. Of those, the patients treated with neoadjuvant alectinib exhibited numerically superior MPR (56.3% vs. 30.8%, *p* = 0.26) and pCR (37.5% vs. 15.4%, *p* = 0.24) rates. OS and PFS were not reached in the alectinib patients, compared to 62.6 months (*p* = 0.226) and 17.9 months for crizotinib (*p* = 0.002), respectively [74]. Single-cell RNA sequencing identified distinct signatures associated with a lack of MPR/pCR (suppressive microenvironment) and a positive pathological response (highly inflamed microenvironment).

The efficacy and safety of ceritinib were evaluated in a small phase 2 trial that enrolled seven patients with stage IIIA *ALK-*rearranged NSCLC [75]. Six out of seven individuals completed 3 × 28-day cycles of TKI therapy—one patient was withdrawn from the study due to hepatitis. The ORR was 100%, with an MPR rate of 57%, and two patients (28.5%) achieved a pCR in this small sample.

The phase II *ALNEO* trial (NCT05015010) investigating neoadjuvant alectinib in resectable stage III ALK-rearranged NSCLC is currently underway and expected to achieve completion in 2026 [76]. Thirty patients have been enrolled and assigned to receive 8 weeks of neoadjuvant alectinib followed by 96 weeks of adjuvant TKI. The primary objective is MPR.

The Lung Cancer Mutation Consortium LCMC4 trial (*LEADER* trial, NCT04712877) is a biomarker-driven precision neoadjuvant screening and matched targeted treatment study for patients with stage IA2-III lung adenocarcinomas and adenosquamous carcinomas. Data from the first 110 patients enrolled were presented in 2024 [77]. Successful genotyping occurred in 91% of the tissue samples submitted (64/70), but only 37% of the blood samples submitted (34/91) had any detectable ct-DNA (defined as tumor fraction > 0). An AGA was identified in 35% (38/110) of the patients, with 100% concordance between blood and tissue results when the ct-DNA was positive. In 8 of 42 patients (19% of cases) with no available tissue for testing, an AGA was present. *EGFR*-sensitizing alterations accounted for 31.5% of the cases, as did *KRAS G12C*. Following the screening results in *LEADER*, the patient may be offered participation in an associated treatment trial, such as *NAUTIKA1*.

*NAUTIKA1* (or ML41591, NCT04302025) is an umbrella, phase 2, multicenter, open-label, and non-randomized trial enrolling patients with resectable stage II, IIIA, or selected IIIB NSCLC with *ALK*, *ROS1*, *NTRK*, and *BRAFV600E* alterations [78]. Pending identification of one of such AGAs, approximately 25 patients per cohort are expected to be treated with alectinib (*ALK*), entrectinib (*ROS1* or *NTRK*), or vemurafenib/cobimetinib (*BRAF V600E*). In the adjuvant phase, the patients are planned to receive four cycles of adjuvant chemotherapy followed by TKIs for up to 2 years. The primary endpoint is an MPR rate, and the secondary objectives include ORR, pCR rate, DFS, EFS, OS, and ct-DNA clearance rate.

### 6.2. Phase III Studies in Neoadjuvant/Perioperative Targeted Therapy

The ongoing *NeoADAURA* trial (NCT04351555) is evaluating neoadjuvant osimertinib with or without chemotherapy versus chemotherapy with a placebo prior to surgical resection in patients with stage II–IIIB N2 *EGFR* mutant + NSCLC, with MPR rates as the primary endpoint [79]. The secondary endpoints include pCR, EFS, OS, and DFS. The trial completed the accrual of 358 patients in October 2024. There are no other phase III randomized controlled studies at the moment that assess neoadjuvant targeted therapy in other AGAs in a 1 L setting.

### 6.3. Phase II Studies in Adjuvant Targeted Therapy 

In the *SELECT* trial, a single-arm study that enrolled a total of 100 patients with resected stage IA-IIIA *EGFR* mutant + NSCLC, the use of adjuvant erlotinib for 2 years led to a 5-year DFS of 56% among the study participants and a 5-year OS of 86% [80]. Ninety per cent of patients experiencing disease recurrence did so after the completion of adjuvant erlotinib therapy. The median time to recurrence after stopping erlotinib was 25 months.

### 6.4. Phase III Studies in Adjuvant Targeted Therapy 

Since the activity of EGFR TKI being restricted to *EGFR* mutant + tumors was not known in the first years after such drugs were developed, a few phase 3 studies evaluating the efficacy of adjuvant EGFR TKI therapy failed to enrich for the correct population. For instance, in the phase III CTG BR19 trial, 505 all-comers with completely resected stage IB-IIIA NSCLC were randomized in a 1:1 fashion to receive gefitinib 250 mg daily or placebo for 2 years. Since 51% of the patients had stage IB disease, only approximately 44% of the patients received adjuvant chemotherapy. No differences in either the OS or DFS were observed between the groups (HR 1.24 and 1.22, respectively) in the ITT population. Disappointedly, the 15 patients with EGFR mutant + NSCLC also did not experience DFS or OS benefit (HR 1.84 and 3.16, respectively) [81].

In retrospect, a similarly flawed study for its enriched population, the *RADIANT* study sought to investigate the role of adjuvant erlotinib versus placebo in patients with tumors demonstrating overexpression of *EGFR* protein or amplification of the *EGFR* gene by FISH. Of note, and again reflecting the extent of scientific knowledge at the time of the trial design, only 16.5% of the ITT population had tumors harboring an *EGFR*-activating mutation. A total of 973 patients with stage IB to IIIA NSCLC (AJCC 6th Edition), *EGFR* mutant + disease, were randomized in a 2:1 fashion to receive either erlotinib or placebo for a total of two years, with the primary endpoint of disease-free survival and a secondary endpoint of overall survival. Approximately 50% of the patients underwent adjuvant chemotherapy and were evenly distributed among the arms. On analysis, no statistically significant difference was observed in DFS between the experimental and placebo group, with a median DFS of 50.5 months in the erlotinib group vs. 48.2 months in the placebo group (HR 0.9; 95% CI, 0.74 to 1.10). A stronger signal favoring erlotinib was observed on subgroup analysis amongst patients with *EGFR*-activating mutations, though this failed to reach statistical significance in great part due to the fact that these patients constituted only 16.5% of the ITT population (*n* = 161 patients) [82]. 

The *IMPACT* trial (WJOG6401L) enrolled and randomized 232 patients with resected stage II–IIIA EGFRm NSCLC in a 1:1 fashion to receive either gefitinib for 2 years or four cycles of cisplatin/vinorelbine. The study did not meet its primary endpoint of DFS, with a numerically superior trend for gefitinib (35.9 months vs. 25.1 months) that failed to show statistical significance (HR: 0.92; 95% CI: 0.67–1.28, *p* = 0.63) [83].

Another randomized phase III trial, *ADJUVANT*-*CTONG*1104, with a similar design and interventions to *IMPACT* (gefitinib × 2 years versus 4 cycles of VP chemotherapy) with 222 patients with resected stage II–IIIA *EGFR*m NSCLC met its primary endpoint of improved DFS for the study arm (28.7 months vs. 18.0 months, HR 0.60, 95% CI; 0.42–0.87; *p* = 0.0054). Its secondary endpoint OS, however, was not different between arms at a median follow-up of 80 months [75.5 vs. 62.8 months, HR 0.92; 95% (CI: 0.62–1.36; *p* = 0.674)] [84].

The role of first-generation *EGFR* TKI icotinib in the adjuvant setting was studied in the Chinese *EVIDENCE* phase III trial enrolling 322 patients with resected stage II–IIIA *EGFR* mutant + NSCLC. Median DFS was 47 months for the group randomized to 2 years of adjuvant icotinib versus 22.1 months for the cisplatin/vinorelbine or pemetrexed arm (HR 0.36; 95% CI 0.24–0.55, *p* < 0.0001). The overall survival data were immature by the time of the publication. Serious treatment-related adverse effects were seen in 1% of the patients treated with TKI versus 14% of the ones assigned to chemotherapy [85].

It was the *ADAURA* trial that established the role of targeted therapy in resected *EGFR* mutant + NSCLC. It examined the efficacy of the third-generation TKI osimertinib by randomizing in a 1:1 fashion a total of 682 patients with resected stage IB (>3 cm but ≤5 cm) to IIIA disease (AJCC 7th Edition) to receive either adjuvant oral osimertinib or a placebo for a total of 3 years, or until disease recurrence or toxicity requiring drug discontinuation. The primary outcome of the DFS and *EGFR* mutation required central confirmation. Adjuvant chemotherapy prior to enrollment in the trial was not required, and 40% of the patients were non-recipients of such. Across the entire cohort, DFS at 24 months among the patients in the osimertinib group was 90%, compared to 44% in the placebo group, with an HR for death or disease recurrence of 0.17 (95% CI, 0.11–0.26). This is suggestive of a staggering 83% decrease in risk of death or disease recurrence for patients receiving adjuvant osimertinib. Moreover, this benefit was sustained in a subgroup analysis across stage IB, stage II, and stage IIIA disease, and amongst both those who received adjuvant chemotherapy and those who did not. For patients with stage IB disease, the DFS at 24 months was 88% in the osimertinib group and 71% in the placebo group, with an HR of 0.39 (95% CI, 0.18–0.76); 91% in the treatment group and 56% in the placebo group amongst patients with stage II disease, with a HR of 0.17 (CI 95%, 0.08–0.31); and 88% in the treatment group and 32% in the placebo group in patients with stage IIIA disease, with a HR of 0.12 (95% CI, 0.07–0.20). Furthermore, among the patients who received adjuvant chemotherapy, the DFS was 89% in the treatment group and 49% in the placebo group, with an HR of 0.16 (95% CI, 0.10–0.26). Likewise, for those who did not receive adjuvant chemotherapy, the DFS in the treatment group was 89% compared to 58% in the placebo group, with an HR of 0.23 (95% CI, 0.13–0.40) [57].

In addition, in the final updated survival analysis of the *ADAURA* study in 2023, a statistically significant difference was demonstrated in overall survival, with an OS at five years of 85% in the treatment group vs. 73% in the placebo group, with a HR for death of 0.49 (95% CI 0.33–0.73) [74]. This pivotal trial established adjuvant osimertinib as the standard of care for patients with resected *EGFR* mutation + NSCLC [72].

To date, the *ALINA* trial is the only phase III randomized controlled trial examining the use of targeted perioperative therapy in patients with resectable, *ALK*-rearranged NSCLC that has published data. In this study, 257 patients with stage IB-IIIA *ALK*-rearranged disease were randomized in a 1:1 fashion to receive adjuvant alectinib for two years or platinum-based chemotherapy. The primary endpoint was DFS, with OS and CNS disease-free survival as secondary endpoints. 

Amongst all patients, the DFS at two years was 93.6% in the alectinib group and 63.7% in the chemotherapy group, with a statistically significant hazard ratio of 0.24 (95% CI, 0.13–0.43), indicating a 76% reduction in disease recurrence or death for patients receiving adjuvant alectinib. Further, the hazard ratio for CNS disease recurrence or death was 0.22, in favor of alectinib (95% CI, 0.08–0.58). The overall survival data were immature at the time of data publication [58].

In the *ALCHEMIST*-ECOG-ACRIN platform A081105 and EA5142 trials, patients with resected stage IB (≥4 cm) to IIIA (AJCC 7th Ed) NSCLC harboring either *EGFR* or *ALK* alterations were assigned adjuvant erlotinib or crizotinib (versus observation), respectively [54]. The results of those studies are not available at the time of this review and may further clarify the impact of such agents in the adjuvant setting.

When comparing the results of both *ADAURA* and *ALINA* to prior trials using earlier-generation, less potent TKIs with less CNS penetration and activity, a couple of questions remain. A very important one refers to the role chemotherapy plays in improving outcomes for these patients. Data stemming from neoadjuvant targeted therapy trials imply that the cytostatic nature of TKI activity may be unable to eradicate micrometastatic disease, and that ongoing TKI suppression may be required for patients with minimal residual disease. Identifying such patients at the greatest risk for systemic relapse is key and the objective of active investigation. In *ADAURA*, the impact of adjuvant osimertinib on both DFS and OS was noted in both recipients and non-recipients of adjuvant chemotherapy. In *ALINA*, however, the patients randomized to alectinib forewent chemotherapy altogether and experienced profound DFS benefit. Both agents prevented CNS relapse remarkably. National guidelines, therefore, reflect the design of those trials and the FDA approval of both drugs recommends adjuvant chemotherapy to patients with stage II–III NSCLC, *EGFR* mutant followed by 3 years of adjuvant osimertinib, whereas they advise to proceed straight to alectinib for 2 years in patients with *ALK* rearranged disease [86] (Table 3 and Table 4).

## 7. Exploring the Role of Radiation in Perioperative NSCLC

The current American Society of Radiation Oncology (ASTRO) Summary of the ASCO Guideline for the Treatment of stage III NSCLC recommends neoadjuvant chemotherapy or chemoradiation for patients with N2 NSCLC who are to undergo surgical resection. The summary guideline also discourages postoperative radiation for completely resected N2 disease, and for patients with unresectable disease, recommends concurrent platinum-based doublet chemotherapy and radiation to a dose of 60 Gy, followed by consolidation durvalumab in patients without progression after initial therapy.

The ASTRO guideline statement reflects the findings of several large prospective randomized controlled trials and meta-analyses of smaller trials that investigated radiation in the neoadjuvant and adjuvant setting alongside systemic therapy and surgery. Prospective randomized controlled trials have not demonstrated an overall survival (OS) benefit with the addition of radiation in the treatment of stage IIIA/N2 NSCLC. Population studies of SEER and NCDB data showed greater OS with postoperative radiation, specifically in subgroups of patients with a larger tumor size, more advanced T stage, more advanced nodal stage, and a pN2 with a greater number of positive nodes according to the SEER analysis.

### 7.1. Preoperative Radiation

The German Lung Cancer Cooperative Group compared induction chemotherapy (cisplatin/etoposide x3) followed by neoadjuvant chemoradiation (45 Gy in 1.5 Gy BID fractions, carboplatin/vindesine) with three neoadjuvant cycles of cisplatin/etoposide and postoperative radiation (54 Gy in 1.8 Gy fractions for R0 resections and 68.4 Gy in 1.8 Gy fractions for R1/2 resections). While the mPFS did not differ between arms, the neoadjuvant chemoradiation arm had greater mediastinal downstaging and pathologic response rates in patients who underwent complete resection [87].

This was followed by the Intergroup 0139 trial comparing resection after neoadjuvant chemoradiotherapy followed by surgery (45 Gy in 1.8 Gy daily fractions with concurrent cisplatin/etoposide) with definitive chemoradiotherapy [88]. At 45 Gy, the patients proceeded to either surgical resection or continued radiotherapy to 61 Gy, with both arms receiving two additional cycles of cisplatin/etoposide consolidation after completion. The primary endpoint of OS did not differ significantly between the two arms. An exploratory analysis suggested that the OS was improved for patients who underwent a lobectomy, but not a pneumonectomy, when compared to definitive chemoradiotherapy.

The ESPATUE trial also compared surgical resection after neoadjuvant chemoradiotherapy (45 Gy in 1.5 Gy BID with cisplatin/paclitaxel) versus definitive chemoradiotherapy (radiation to a total of 65–71 Gy with concurrent cisplatin/vinorelbine) [89]. ESPATUE was terminated early due to slow accrual and therefore lacked the statistical power to evaluate its endpoints. However, similar 5 yr OS and PFS were reported for each arm.

The Swiss Group for Clinical Cancer Research (SAKK) 16/00 trial investigated the addition of preoperative radiation (44 Gy in 2 Gy fractions) to neoadjuvant chemotherapy (cisplatin/docetaxel x3) prior to surgery [90]. Postoperative radiation was given to patients with R1/R2 resections in the chemotherapy-only arm. The study was not powered for OS, but the addition of preoperative radiation did not improve OS or EFS.

### 7.2. Postoperative Radiation

The ANITA trial, discussed in the Introduction, randomized patients with completely resected NSCLC stages IB to IIIA to adjuvant chemotherapy (cisplatin/vinorelbine). The use of postoperative radiation therapy (PORT) was recommended for pN + disease but was not randomized or mandatory. An unplanned subgroup analysis reported greater survival in patients with pN2 disease who received postoperative radiotherapy in both the chemotherapy and observation arms [91]. In patients with pN1 disease, survival was greater with PORT in the observation arm but lower in the chemotherapy arm.

The PORT-C trial evaluated the addition of postoperative radiation (≤50 Gy/2 Gy per fraction regimens were permitted) following surgery and adjuvant chemotherapy (platinum-based x4 cycles) [92]. Notably, the radiotherapy arm had a 24% noncompliance rate. The intention-to-treat analysis showed a 3-year local control benefit in the radiotherapy arm. Although an intention-to-treat analysis did not show a 3-year OS or DFS benefit with the addition of radiotherapy, the per-protocol analysis showed a DFS benefit.

Lastly, the Lung ART trial also evaluated the addition of postoperative radiation (54 Gy/27–30 fx) following surgery but allowed neoadjuvant or adjuvant chemotherapy [93]. No DFS or OS benefit was shown. The radiotherapy arm had a lower rate of mediastinal recurrence and death from progression, but a higher rate of deaths from toxicity. Ninety percent of the radiation plans were 3D conformal, as opposed to contemporary IMRT planning. It should be noted that none of these trials have included the use of checkpoint inhibitor therapy. However, that is further examined in later sections.

Despite the lack of consensus regarding the role of postoperative radiotherapy in resected locally advanced NSCLC, the American Society for Radiation Oncology nonetheless provided guidance that emphasized the need to consider postoperative radiation in patients at higher risk of locoregional recurrence (R1 and R2 resections).

## 8. Management of N2 Disease

The surgical treatment of patients with an involvement of ipsilateral mediastinal and/or subcarinal nodal stations has always been a matter of debate and subject to institutional practices, experience, and expertise. It certainly demands a multimodality approach, and previously, many thoracic surgeons would elect for chemoradiotherapy in most cases due to a very high risk of systemic relapse. The driving clinical decision points in determining NSCLC resectability include lymph node volume (“bulkiness”), involvement of single vs. multiple LN stations, and the size and resectability of the actual primary tumor (stage IIIA vs. IIIB disease). In a 2021 review of European thoracic surgeons, it was demonstrated that surgeons are more frequently opting for the surgical management of non-bulky, single-station N2 disease [20]. This reflects the recognition that not all N2 diseases are created the same, with the most recent AJCC 9th Ed. classification stratifying N2 staging according to the number of stations involved, as this impacts long-term survival [18]. While direct studies examining the role of neoadjuvant immunotherapy in tumor bulk reduction specifically in N2 disease have yet to be completed, a subgroup analysis of the perioperative chemo-immunotherapy trial *Checkmate 77T* by nodal demonstrated that, of patients with N2 disease receiving perioperative nivolumab, 46% underwent nodal downstaging to ypN0 compared to 36% in the placebo arm. While the OS data is still immature, the EFS data was also improved, with a median EFS rate in the stage III N2 disease nivolumab arm not reached (95% CI, 23.8-NR) compared to 8.9 months in the placebo arm (95% CI, 6.1–15.6) [94]. We believe the evidence from the neoadjuvant/perioperative immunotherapy trials discussed here, which demonstrate a benefit in EFS and, more specifically, in pathological complete response, supports a role for immunotherapy in further expanding our definition of resectable N2 disease by improving pre-operative nodal downstaging and post-operative disease recurrence. 

As it currently stands, there are several active phase II trials exploring this particular question. The *ESPADURVA* trial, currently slated to be completed by April of 2025, is investigating the efficacy and safety of adding neoadjuvant durvalumab to standard neoadjuvant chemoradiation therapy in patients with stage III NSCLC [95]. A number of other early-phase trials, such as *SAKK 16*/*18*, are currently investigating the role of the induction of concurrent radiation therapy alongside neoadjuvant chemo/immunotherapy in patients with stage III disease [96]. That said, much investigation remains to be conducted in determining the most effective management of stage III disease. 

Nevertheless, it is imperative that systematic mediastinal staging of such patients, preferably by minimally invasive procedures such as bronchoscopy with endobronchial ultrasound (EBUS) aid be performed in all patients with clinical stage IB and above. FDG avidity or lack thereof in PET scans should not be taken as final staging when making treatment decisions in this population.

## 9. Future Directions

### 9.1. Innovative Approaches to Perioperative Management

The future landscape of adjuvant and neoadjuvant/perioperative treatment in NSCLC is expanding in all directions. The phase II *NeoCOAST* trial, presented at the American Association of Cancer Research Annual Meeting in 2022, serves as an example. This open-label, multidrug platform study enrolled stage I (>2 cm)-IIIA resectable NSCLC patients, utilizing a durvalumab backbone neoadjuvantly with the possible addition of a second investigation agent. The agents added to durvalumab in this basket study included an anti-CD73 monoclonal antibody (mAb), oleclumab, an antiNKG2A mAb, monalizumab, or an anti-STAT3 antisense oligonucleotide, danvatirsen. A preliminary analysis demonstrated improved MPR and pCR rates. MPR occurred in 11.1% with durvalumab, 19.0% with additional oleclumab, 30.0% with additional monalizumab, and 31.3% with additional danvatirsen. A pCR occurred in 3.7%, 9.5%, 10.0%, and 12.5% (95% CI 1.6–38.3), respectively. While this data is still preliminary, it is encouraging and represents the ever-expanding treatment paradigm in perioperative NSCLC [97].

The *TOP 2301* (NCT06385262) phase II trial is looking at combining a PCSK9 inhibitor (alirocumab) with chemotherapy and the PD-1 inhibitor cemiplimab in patients with stage IB-IIIA NSCLC. The pre-clinical data have demonstrated that, by inhibiting PCSK9, a protein involved in cholesterol metabolism, we may enhance the recruitment of CD8 + T cells and reduce the numbers of Tregs, enhancing immune response [98].

Another very interesting approach is to enhance the efficacy of adjuvant checkpoint inhibitor therapy by combining it with personalized neoantigen therapy. In the *INTerpath-002* trial (NCT06077760), the mRNA-4157 (V940) vaccine is constructed by sequencing the patient’s tumor obtained during surgical resection to identify up to 34 unique neoantigens and administered with pembrolizumab (versus placebo + pembrolizumab) in patients with resected stage II–IIIB (N2) NSCLC after the completion of adjuvant chemotherapy. *INTerpath-009* will explore this approach in patients who do not achieve pCR after undergoing neoadjuvant chemoimmunotherapy. The *KEYNOTE-942* study in patients with resected stage IIIB-IV high-risk melanoma demonstrated a 49% decreased risk of recurrence or death compared to pembrolizumab alone (HR 0.56; 95% CI: 0.309–1.017, *p* = 0.053) [99].

### 9.2. Risk Stratification and Personalized Care: Exploring the Role of ctDNA in NSCLC Treatment Planning and Surveillance

As we presented previously, the systemic therapy landscape of resected/resectable NSCLC has changed dramatically over the last few years. However, although clinical and pathological staging are key predictors of systemic relapse, a vast gap remains in determining precisely who benefits from escalated additional therapy. How can we identify patients bound to achieve a survival benefit from receiving additional adjuvant therapy after a neoadjuvant course or immunotherapy after undergoing adjuvant chemotherapy? One method that can help distinguish high-risk from low-risk patients after surgery, regardless of stage, is circulating tumor DNA (ctDNA) detection and measurement.

Of particular interest is the *MERMAID-2* trial, which enrolls patients with stage II–III NSCLC treated with curative intent therapy (optional neoadjuvant and/or adjuvant therapy) to 96 weeks of ctDNA surveillance. It is testing a personalized (or tumor-informed) minimal residual disease (MRD) panel developed using resection specimens. If/when minimal residual disease (MRD) is detected, the patients are randomized 1:1 to up to 24 months with durvalumab or placebo. While the data have not been reported at the time of writing, this trial is particularly noteworthy, as it is the first that bypasses chemotherapy altogether at MRD detection [100].

As the larger trials continue to mature, additional data and exploratory endpoints are reported. At the European Society of Medical Oncology (ESMO) 2022 conference, *IMPower010* reported a median DFS improvement of 18 months in favor of atezolizumab in those who cleared ctDNA after chemotherapy [101]. ESMO 2024 brought additional data, including IMPower010-reported median OS data stratified by ctDNA positivity. Patients with post-operative ctDNA clearance did not reach median OS in either the experimental atezolizumab arm or the BSC arm. For those ctDNA positive post-operatively, the median OS was 68.5 months in the atezolizumab arm compared to 32.4 months in the BSC arm (HR 0.58, 95% CI 0.32–1.05), suggesting another way ctDNA could help guide the decision to initiate adjuvant immunotherapy [102].

However, many challenges remain with regard to this approach, including the sensitivity and the specificity of the testing platform, standardization (tumor-informed versus tumor-naïve assays), and the optimal timing for testing.

## 10. Conclusions

The landscape of systemic treatment in resectable NSCLC has changed drastically over the past 5 years with the introduction of checkpoint inhibitors and targeted therapies. Several ground-breaking studies with such therapies have demonstrated remarkable survival benefits for patients, compared to standard chemotherapy alone. Novel immunotherapy and combination trials are ongoing, while several studies are further investigating the role of immunotherapy in a multimodality approach to effectively treat more advanced stage III N2 diseases that were previously considered unresectable. 

Despite these exciting advances, much remains to be learned about how to best individualize the sequencing of effective regimens, as well as the treatment duration/length. The need to minimize long-term toxicities in patients treated with curative intent is imperative.

## Figures and Tables

**Table 1:** Definition and utility of surrogate endpoints for overall survival.

Surrogate Endpoint	Definition	Utility	Limitations
Disease-Free Survival (DFS) [25,26]	Time from surgery or randomization to the first recurrence of cancer or death from any cause	Frequently used in adjuvant trials; accepted by FDA for approval of new therapies in NSCLC	Does not always correlate with OS, as some patients who relapse can still achieve long-term survival with additional therapies
Event-Free Survival (EFS) [27]	Time from randomization to disease progression, first recurrence of cancer, abandoned surgery, or death from any cause prior to surgery	Often used in neoadjuvant trials; also reflects impact of neoadjuvant therapy on ability to take patient to surgery	Similar to DFS, may not correlate with OS in patients who can be salvaged after recurrence with additional treatment
Pathologic Complete Response (pCR) [28,29,30]	Absence of any residual viable tumor cells in resected lung and lymph nodes following neoadjuvant treatment	Surrogate in neoadjuvant studies has shown strong correlation with lower risk of recurrence and better long-term survival; accepted by FDA for approval of new therapies in NSCLC	Variability in definition across studies, inconsistent correlation with OS, fails to capture long-term nuances of treatment response
Major Pathologic Response (MPR) [31,32]	≤10% of residual viable tumor cells in primary tumor following neoadjuvant treatment	Strong correlation with long-term outcomes in neoadjuvant studies; may be more sensitive than pCR	Inconsistent correlation with OS, fails to capture long-term nuances of treatment response
Recurrence-Free Survival (RFS) [33]	Time from treatment to first occurrence of cancer recurrence, not including death from other causes	Useful in cases where death from other causes might confound survival analysis	Does not directly correlate with overall survival because it excludes death from other causes
Progression-Free Survival (PFS) [33]	Time from the start of treatment until disease progresses or patient dies from any cause	Useful in both adjuvant and neoadjuvant studies as an early signal of treatment efficacy	May not correlate well with OS amongst patients for whom subsequent treatments are available after progression
Minimal Residual Disease (MRD) [34,35,36]	Refers to presence/absence of small numbers of detectable, circulating cancer cells after treatment (i.e., molecular DNA or circulating tumor DNA assays)	Associated with better long-term outcomes, as it indicates near complete eradication of all circulating tumor cells	Still under investigation as a reliable surrogate for OS in NSCLC
Time to Recurrence (TTR) [37]	Time from surgery to the recurrence of NSCLC; excludes death from other causes	Similar to RFS, can help isolate effects of treatment on tumor biology without the confounding variable of unrelated deaths	Does not directly correlate with overall survival because it excludes death from other causes

**Table 2:** Select neoadjuvant/perioperative immunotherapy trials.

Study Name	NCT #	Type of Study	Treatment/Arms	Number of Participants	OS (Study/Placebo)	DFS/PFS/EFS (Study/Placebo)	MPR (Study/Placebo)	pCR (Study/Placebo)
Forde et al. [46]	02259621	Pilot study Single Arm	Neoadj. pembrolizumab	21	-	DFS 73% (at 18 months)	45%/NA	9.5%/NA
Eichhorn et al. [39]	03197467	Pilot study Single Arm	Neoadj. pembrolizumab	15	-	-	27%/NA	-
LCMC3 trial [40]	02927301	Phase II Single Arm	Neoadj. atezolizumab	181	-	-	20%/NA	-
NEOSTAR trial [41]	03158129	Phase II Randomized	Neoadj. nivolumab or neoadj. nivolumab + ipilimumab	44	-	-	38% (nivolumab + ipilimumab) 22% (nivolumab monotherapy)	38% (nivolumab + ipilimumab) 10% (nivolumab monotherapy)
Shu et al. [42]	02716038	Phase II Single Arm	Neoadj. atezolizumab w/neoadj. carboplatin/nab-paclitaxel	30	-	-	57%/NA	33%/NA
NADIM trial [42,43]	03081689	Phase II Single Arm	Neoadj. nivolumab in combination with carboplatin/paclitaxel, followed by adjuvant nivolumab for 6 months	46	90%	PFS 77% (at 24 months)	83%/NA	63%/NA
NADIM II trial [44]	03838159	Phase II Randomized	Neoadj. nivolumab plus carboplatin/paclitaxel vs. chemotherapy alone	86	85%/63.6% (at 24 months) HR for death 0.43 (95% CI, 0.19–0.98) *	PFS 67.2%/40.9% (at 24 months) HR 0.47 (95% CI, 0.25–0.88) *	-	37%/7% *
CheckMate 816 [45]	02998528	Phase III Randomized	Neoadj. nivolumab plus carboplatin/paclitaxel vs. chemotherapy alone	358	-	EFS 31.6 mo/20.8 mo HR 0.63 (95% CI, 0.43 to 0.9) *	-	24%/2.2% *
KEYNOTE 671 [48]	03425643	Phase III Randomized	Neoadj. pembrolizumab plus platinum-based therapy, followed by adj. pembrolizumab vs. neoadj. chemotherapy alone and adj. placebo	397	80.9%/77.6% (at 24 months)	EFS 62.4%/40.6% (at 24 months) HR 0.58 (95% CI, 0.46–0.72) *	30.2%/11% *	18.1%/4% *
AEGEAN [49]	03800134	Phase III Randomized	Neoadj. durvalumab plus platinum-based therapy, followed by adj. durvalumab vs. neoadj. chemotherapy alone with adj. placebo	802	-	EFS 73.4%/64.5% (at 12 months) HR 0.68 (95% CI, 0.53 to 0.88) *	-	17.2%/4.3% *
CheckMate 77T [47]	04025879	Phase III Randomized	Neoadj. nivolumab plus platinum-based chemotherapy followed by adj. nivolumab for 1 year vs. neoadj. chemotherapy alone plus adj. placebo	Ongoing enrollment	-	EFS 70.2%/50% (at 18 months) HR 0.58 (95% CI, 0.42 to 0.81) *	35.4%/12.1% *	25.3%/4.7% *
NEOTORCH trial [50]	04158440	Phase III Randomized	Neoadj. toripalumab plus platinum-based chemotherapy followed by adjuvant torpalumab vs. neoadj. chemotherapy alone and adj. placebo	501	-	EFS non-estimable/15.1 HR 0.4 (95% CI, 0.28–0.57) *	48.5%/8.4% *	24.8%/1% *

* *p* < 0.05, NA = Not Applicable.

**Table 3:** Select neoadjuvant/perioperative targeted therapy trials.

Study Name	NCT #	Type of Study	Targeted Mutation	Treatment/Arms	Number of Participants	ORR	OS (Study/Placebo)	DFS/PFS/EFS (Study/Placebo)	MPR (Study/Placebo)	pCR (Study/Placebo)
Lara-Guerra et al. [63]	00188617	Phase II Single Arm	*EGFR*	Neoadj. gefitinib	36	11%/NA	-	-	-	-
Zhong et al. [64]	00600587	Phase II	*EGFR*	Neoadj. Erlotinib vs. gemctabine/cisiplatin	24	58.3%/25%	14.5 months/28.1 months	PFS 6.9 months/9.0 months	-	-
Zhang, Y et al. [65]	01833572	Phase II Single Arm	*EGFR*	Neoadj. Gefitinib	33	54.5%/NA	-	DFS 33.5 months/NA	24.2%/NA	-
Xiong et al. [66]	01217619	Phase II Single Arm	*EGFR*	Neoadj. erlotinib	15	67%/NA	51 months/NA	-	-	67%/NA
ML25444 trial (continuation of Xiong et al.) [67]	01217619	Phase II Single Arm	*EGFR*	Neoadj. erlotinib	19/NA	42.1%/NA	51.6 months/NA	PFS 11.2 months/NA	-	-
EMERGING trial [69]	01407822	Phase II Randomized	*EGFR*	Neoadj + adj. erlotinib vs. Neoadj. + adj. gemcitabine/cisplatin	386	54.1%/34.3%	-	PFS 21.5 months/11.4 months HR 0.39 (95% CI, 0.23 to 0.67) *	9.7%/0%	Not observed in either arm
Bian et al. [70]	04201756	Phase II Single-arm	*EGFR*	Neoadj. afatinib	47	70.2%/NA	Not reached	Not reached	9.1%/NA	3.0%/NA
CTONG1103 [84] trial (continuation of EMERGING trial)	01407822	Phase II Randomized	*EGFR*	Neoadj. + adj. erlotinib vs. neoadj./adj gemcitabine/cisplatin	386	-	42.2 months/36.9 months	-	-	-
NEOS trial [71]	ChiCTR1800016948	Phase II Single Arm	*EGFR*	Neoadj. osimertinib	38	7.1%/NA	-	-	-	-
Blakely et al. [72]	03433469	Phase II Single Arm	*EGFR*	Neoadj. osermitinb	27	52%/NA	-	DFS 40.9/NA	14.8%/NA	None observed
SAKULA trial [75]	UMIN000017906	Phase II Single Arm	*ALK*	Neoadj. ceritinib	19	100%/NA	-	-	57%/NA	28.6%/NA
ALNEO tria [76]	EUDRACT 2020-003432-25	Phase II Single Arm	*ALK*	Neoadj. alectinib	30	In process	In process	In process	In process	In process
Blakely et al. [72]	03433469	Phase II Single Arm	EGFR	Neoadj. osermitinib	27	52%/NA	-	DFS 40.9/NA	14.8%/NA	0%/NA
ML41591/Nautika1 [78]	04302025	Phase II	*ALK*, *ROS1*, *NTRK*, *BRAFV600*	Neoadj. Plus adj. Alectinib (ALK), entrectinib (ROS1 and NTRK), vemurafenib plus cobimetinib (BRAF600)	100	In process	In process	In process	In process	In process
Zhang, C et al. [73]	-	Case report Single Arm	*ALK*	Neoadj. Crizotinib	11	90.9%/NA	-	-	-	18.2%/NA
Zhang, C et al. [74]	-	Case report Double Arm	*ALK*	Neoadj. Crizotinib vs. neoadj alectinib	29	73.7 (crizotinib)/71.4% (alectinib)	62.2 months (crizotinib)/not reached (alectinib)	PFS 17.9 months (crizotinib)/not reached (alectinib group)	46.2% (crizotinib)/64.7% (alectinib)	15.4% (crizotinib)/35.3% (alectinib)
NeoADAURA [79]	4351555	Phase III	*EGFR*	Neoadj. Osermitinb +/− chemotherapy vs. chemotherapy alone	358	In process	In process	In process	In process	In process

* *p* < 0.05.

**Table 4:** Select adjuvant targeted therapy trials.

Study Name	NCT #	Type of Study	Targeted Mutation	Treatment/Arms	Number of Participants	ORR	OS (Study/Placebo)	DFS/PFS/EFS (Study/Placebo)
SELECT trial [80]	00462995	Phase II Single Arm	*EGFR*	Adj. Osimertinib	100	-	86% (after 5 years)/NA	DFS (after 5 years) 88%/NA
NCIC CTG BR19 trial [81]	00049543	Phase III Randomized	*EGFR*	Adj. Gefinib vs. placebo	503	-	5.1 years/not reached	4.2 years/not reached
RADIANT trial [82]	00373425	Phase III Randomized	*EGFR*	Adj. Erlotinib vs. placebo	973	-	Data immature	DFS 50.5/48.2
ADAURA trial [57]	02511106	Phase III Randomized	*EGFR*	Adj. Osimertinib vs. placebo	682	-	85%/73% (after 5 years) ** HR 0.49 (95% CI, 0.33 to 0.73) *	DFS 90%/44% (at 24 months) HR 0.17 (95% CI, 0.11 to 0.26) *
IMPACT trial [83]	UMIN000006252	Phase III Randomized	*EGFR*	Adj. gefitinib vs. cisplatin plus vinorelbine	232	-	78%/74.6%	DFS 35.9/25.1
EVIDENCE trial [85]	02448797	Phase III Randomized	*EGFR*	Adj. Icotinib vs. platin-based chemotherapy	322	-	Data immature	Median DFS 44 months/22.1 months HR 0.36 (95% CI, 0.24–0.55) *

* *p* < 0.05, ** Not measured as an initial primary outcome; evaluated as part of final updated analysis in 2023.
